# Assessing Tick-Borne Disease Risk and Surveillance: Toward a Multi-Modal Approach to Diagnostic Positioning and Prediction

**DOI:** 10.3390/microorganisms10040832

**Published:** 2022-04-18

**Authors:** Chris Brooks, Connie L. McNeely, Sarah P. Maxwell, Kevin C. Thomas

**Affiliations:** 1 Laboratory for Human Neurobiology, Boston University School of Medicine, Boston, MA 02118, USA; crbrooks@bu.edu (C.B.); kipthoma@bu.edu (K.C.T.); 2 Center for Science, Technology, and Innovation Policy, George Mason University, Fairfax, VA 22030, USA; cmcneely@gmu.edu; 3 School of Economic, Political & Policy Sciences, University of Texas at Dallas, Richardson, TX 75080, USA

**Keywords:** tick-borne disease, disease surveillance, Lyme disease, entomology, canine serologic, tick bite encounter

## Abstract

The true extent of tick-borne disease (TBD) incidence and risk among humans is largely unknown, posing significant public health challenges. This study offers an exploratory analysis of a multimodal dataset and is part of a larger ongoing project to determine if entomological data, canine serological reports, self-reported human tick bite encounters (TBEs), and/or associated TBD diagnoses can serve as proxies for human disease risk. Focusing on the United States (U.S.), it characterizes self-reported TBD diagnoses (specifically, anaplasmosis, ehrlichiosis, and Lyme disease), co-infections, and their frequency and distribution across U.S. counties in relation to the presence of other factors related to TBD risk. Survey data was used to construct a list of TBEs localizable to individual U.S. counties. National data regarding these counties—namely the presence of official Lyme Disease (LD) case reports from the Centers for Disease Control and Prevention, as well as the tick vectors *I. scapularis* and *I. pacificus* within a given county—were then linked with survey-reported TBEs, tabulated by diagnosis (including co-infections), to determine the distribution of county-level endpoints across diagnostic categories. In addition, data on the presence of positive serological diagnostic tests conducted in canines were considered due to their potential utility as a proxy for TBD and TBE risk. The final dataset contained 249 TBEs localized to a total of 144 counties across 30 states. Diagnostic categories included respondents with LD (*n* = 70) and those with anaplasmosis and ehrlichiosis diagnoses and co-infections (*n* < 20 per diagnostic category). TBEs also were indicated by respondents who did not report TBD diagnoses, with some indicating uncertainty. The distribution of respondent-reported TBEs varied between canine TBDs, with LD-positive respondents reporting noticeably larger proportions of TBEs in counties with canine LD and smaller proportions in counties with canine anaplasmosis, compared to respondents without an LD diagnosis; a notional logistic regression suggests these differences may be significant (canine LD: Odds Ratio [OR] = 6.04, *p* = 0.026) (canine anaplasmosis: OR = 0.50, *p* = 0.095). These results suggest that certain widely available diagnostic TBD data in animals (in this case, domesticated dogs) may be sensitive to differences in human TBD risk factors and thus may have utility as proxies in future research. In the absence of an available standardized, unified, and national TBD database, such proxies, along with relevant surveys and reports, may provide a much-needed working solution for scientists and clinicians studying TBDs.

## 1. Introduction

The increasing geographic range of tick presence and of tick-borne disease (TBD) cases has been accompanied by growing concerns regarding diagnostic challenges, treatments, and overall effects. Although they appear and are increasing in presence in countries around the world [1,2], TBDs (e.g., Lyme borreliosis, anaplasmosis, babesiosis, ehrlichiosis, and spotted fever rickettsiosis, among others) are significantly more widespread in the United States (U.S.) [3], where they are becoming an increasingly pervasive public health problem.

Of particular note in this regard is the increase in Lyme disease (LD). More than 476,000 new LD cases have been estimated per year in the U.S. [4], and that figure is expected to increase by at least 20% over the next 10–20 years [5]. Moreover, while LD represents the greatest growth, there appears to be an expanding distribution of TBDs in general, driven largely by accelerated tick population increases in response to environmental factors and climate change impacts on their life cycles, especially where warmer conditions prevail [1,6]. In fact, the potential range of tick locations may far exceed current official endemic designations [7]. Accordingly, the need to assess ecosystem and spatial variations in infected vector density is receiving particular attention in research aimed at determining change in TBD risk patterns among humans [7,8].

As is the case with other vector-borne diseases, research on the risk and health impacts of TBDs is complicated by challenges with conflicting medical viewpoints, surveillance, imprecise diagnostic tools, incomplete solutions, and variable symptomology [9]. Among other primary concerns that arise in regard to TBDs are symptom misattribution and/or secondary infection vulnerability. TBDs may present with a wide range of symptom variations, making them difficult to diagnose and often leading to mistaken identification of other diseases. For example, chronic LD in particular is “a multisystem illness with diverse musculoskeletal, neuropsychiatric, and/or cardiovascular manifestations” often associated with other TBD pathogens [10]. TBDs have been linked to an array of autoimmune and immune-mediated diseases, as well as inflammatory disorders, and secondary infections exacerbate the suffering of those with related primary disorders conditions. Co-infection—referring to simultaneous infection with two or more pathogens within the same vector or host [8]—contributes to illness severity and is a common problem associated with TBDs. The association of other microorganisms in relation to TBDs are critical determinants in terms of disease severity, disease length, health complications and adverse effects, need for treatment, hospitalization, and healthcare costs. Faced with such issues, there is a pressing need for research that not only attends to TBD incidence, but also to broader implications for disease susceptibility, progression, and prognosis [11].

This study follows other research on TBD surveillance showing that comparisons between patient self-reported disease and official counts of disease can be important epidemiological tools when disease can be linked to an event, such as a tick bite, in relation to locational indicators at different geographical levels of analysis and ecosystem characteristics [7]. Accordingly, locational and ecosystem data are integrated with national TBD epidemiological data and other available patient reports and surveys on tick bite encounters, related diagnoses, and symptoms. They are incorporated in contextually sensitive analyses to contribute to the general knowledge base and to inform public health policy responses. The research emphasizes the importance of finer grained locational and eco-regional analyses in relation to various TBD indicators and factors to improve disease surveillance and understanding of disease risk. Since aspects of LD and other TBDs vary geographically—including “the primary causative agents and their vectors; pathogenicity and common disease manifestations; and the prevalence and incidence of disease” [12]—spatial considerations are a critical binding feature of this study. Indeed, looking just at those transmitted by I. scapularis, seven microorganisms are known to cause illness in humans [8]: five bacteria (*Anaplasma phagocytophilum*, *Bo. burgdorferi*, *Bo. mayonii*, *Bo. miyamotoi*, and *E. muris eauclarensis*), one protozoan parasite (*Babesia microti*), and one virus (Powassan virus). Additionally, another analytical factor is based on the fact that ticks on “companion animals,” such as dogs and cats, are a significant risk factor for spreading related pathogens, posing a threat to humans with regard to TBD transmission [1,8]. Thus, available veterinary data on canine tick disease is used as a proxy for assessing tick presence and density potentially affecting human TBD risk.

In a broader sense, while this analysis is exploratory, focusing on associations between tick prevalence and disease risk, it also points to directions for expanded and more encompassing research in the future.

## 2. Materials and Methods

Analysis focused on describing the distribution, overlap, and cross-tabulation of multimodal endpoints, collected at the individual and county level, related to tick bite encounters (TBEs) and tick-borne disease (TBDs). The main endpoints and variables of interest were self-reported TBEs and TBD diagnoses (specifically, Lyme Disease [LD], anaplasmosis, and ehrlichiosis) obtained via online survey, positive tests for these same TBDs in canines obtained from the Companion Animal Parasite Council (CAPC), and the presence of tick vectors and human LD cases as officially reported by the CDC.

### 2.1. Survey and Respondent Data

Starting in 2020, the Tick Bite Encounter Survey (henceforth referred to as the “survey”) was distributed online via selected national LD-related nonprofit organization websites (e.g., Global Lyme Alliance) and social media pages (e.g., Facebook) to attract individuals diagnosed with a tick-borne illness. The survey was made available using a shareable link, and respondents were allowed to freely disseminate the link on social media in order to reach as large a population as possible. The survey was available for fifteen months (1 January 2020 through 31 March 2021) via an anonymous link (administered by Qualtrics). Participation was voluntary and required the respondent’s consent for access. Respondents provided information about their TBEs, diagnosed TBDs (i.e., LD, anaplasmosis, and ehrlichiosis), symptoms, and interactions with—as well as perceptions of—the medical community. The survey was anonymous and confidential; researchers did not request or receive any identifying personal data.

### 2.2. National County-Level Data

National data were derived from two main sources: (1) Official US by-county databases maintained by the CDC. These included total number of human LD cases that met CDC diagnostic criteria (see above) and were recorded by the CDC between the years of 2000 and 2019; and tick presence, specifically counties officially established and reported by the CDC to contain *I. scapularis* ticks as of 2020 and counties officially established and reported by the CDC to contain *I. pacificus* ticks as of 2020 [13]; (2) CAPC county-level databases, including the following: total number of serological tests conducted on canines in 2020; number of these tests positive for ehrlichiosis, anaplasmosis, and Lyme Disease [14].

### 2.3. Database and Data Alignment

To maximize the value of the covariate data (e.g., presence of CDC-positive LD cases), only TBEs that occurred in the U.S. between 1 January 2000 and 31 March 2021 were included in the analysis. In addition, TBEs were only analyzed if the location provided by the respondent could be unambiguously localized to a single zip code or county, which was used to link self-reported survey data (TBEs, TBD diagnoses, and respondent characteristics) to county-level data from national sources (CAPC and CDC data). The data were organized so that each observation in the sample (i.e., unit of analysis) represented a single TBE reported by a single survey respondent, including the county the TBE occurred in and whether the respondent reported a diagnosis of LD, anaplasmosis, ehrlichiosis, or any combination (i.e., co-infection) of these TBDs; note that, while self-reported diagnostic data was collected for other diseases as well (e.g., babesiosis), the co-infections analyzed in this study are limited to just these three TBDs.

County-level data obtained from national databases were linked with respondent survey TBE data via the county the TBE occurred in. The raw frequencies were converted into dichotomous dummy variables (e.g., “Did ____ county have 0 CDC LD cases, or 1+ CDC LD cases?”) representing the presence (or absence) of each of the county-level endpoints, specifically the following:

− Presence of at least 1 CDC-reported LD case between the years of 2000 and 2019;
− CDC-reported presence of *I. scapularis* and/or *I. pacificus* as of 2020;
− Presence of at least 1 CAPC-reported canine anaplasmosis case in 2020;
− Presence of at least 1 CAPC-reported canine ehrlichiosis case in 2020;
− Presence of at least 1 CAPC-reported canine LD case in 2020.

In summary, each observation consisted of a single survey-reported TBE, participant-level diagnostic and demographic data from the respondent who reported the TBE, and county-level epidemiological dummy data of the county the TBE was reported in.

Descriptive statistics were generated for all analytical variables. Unless otherwise specified, continuous variables and percentages were summarized using median (inter-quartile range), and categorical variables were summarized using count (percentage). Data used in analysis were organized and arrayed using Excel 16.52 for Mac (Microsoft, Inc., Redmond, WA, USA). All statistical analyses were performed in Stata/SE 16.1 for Mac (StataCorp, Inc., College Station, TX, USA). A p-value less than 0.05 was considered statistically significant, and a *p*-value greater or equal to 0.05 and less than 0.10 was considered approaching significance.

## 3. Results

A total of 239 respondents completed the online survey before the predetermined cut-off date of 31 March 2021. Of these, 181 (75.73%) respondents reported at least one TBE with a combined total of 329 individual TBEs reported, with an average (standard deviation) of 1.82 (1.11) and a median or interquartile range of 1 (1) TBE per “TBE-positive” respondent. Note that the design of the digital survey prevented respondents from reporting more than 4 individual TBEs. The distribution of respondent characteristics between those who did and did not report a TBE are presented in Table 1. TBEs were filtered to restrict the sample only to those who provided sufficient data for analysis. The final analysis dataset consisted of 249 (75.68%) TBEs across 148 respondents (61.92% of all respondents, 81.77% of TBE-positive respondents), as shown in Table 2. These were spread over 144 U.S. counties and, unless otherwise noted, all county-level proportions and percentages described herein were derived from this set of 144 counties; likewise, all respondent-level statistics (e.g., self-reported diagnoses) were derived from the final analytical cohort of 148 respondents. Note that 15 of the 249 (6.02%) TBEs occurred in counties with no CAPC data (no TBEs occurred in counties with missing CDC data); because the absence of canine TBDs could not be verified via the CAPC, the 15 TBEs in these counties were omitted from all summary statistics of canine TBDs. Therefore, results involving canine TBDs have *n* = 234 TBEs, whereas results involving CDC LD cases or tick presence have *n* = 249 TBEs.

Simple frequencies were generated for all county-level variables within groups determined by self-reported TBD diagnoses of anaplasmosis, ehrlichiosis, LD, and any combination of these (co-infections). Frequencies and percentages of nationally sourced endpoints in counties with at least one survey-reported TBE, cross-tabulated by self-reported diagnosis of anaplasmosis, ehrlichiosis, and/or LD, are presented in Table 3A,B.

### 3.1. Distribution of Self-Reported Infections and Co-Infections

**Figure 1:**
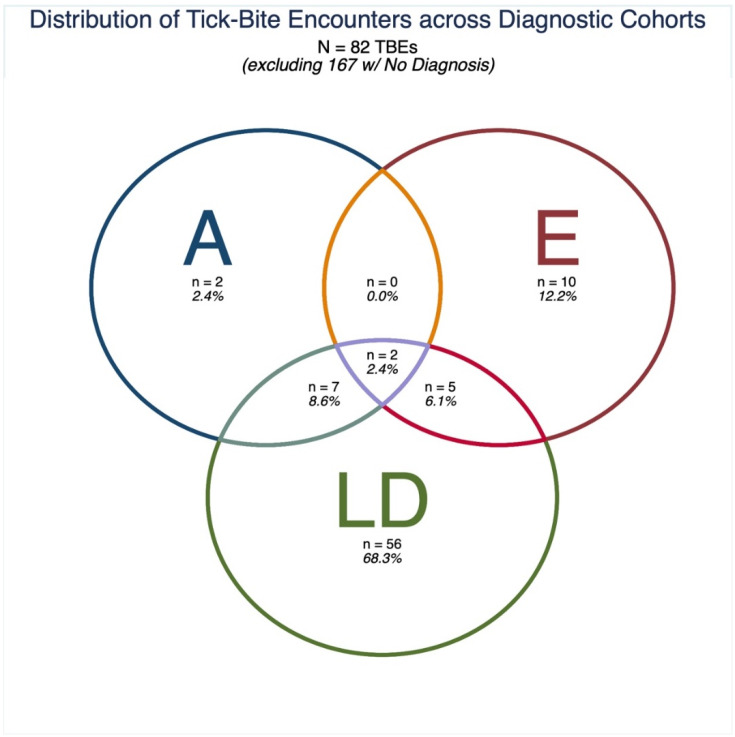
Distribution of tick-bite encounters (TBEs) across diagnostic cohorts, excluding TBEs from respondents who did not report a tick-borne disease (TBD) diagnosis. Anaplasmosis (A), ehrlichiosis (E), or Lyme Disease (LD).

Out of the final analytical cohort of 148 respondents (Table 2), 97 (65.54%) reported no diagnosis of LD, anaplasmosis, or ehrlichiosis (note: some survey respondents also responded “maybe/I don’t know,” indicating they may have taken the survey unsure of the cause of an undiagnosed illness); 34 (22.97%) reported a diagnosis of just LD; 6 (4.05%) reported a diagnosis of just ehrlichiosis; 2 (1.35%) reported a diagnosis of just anaplasmosis; 3 (2.03%) reported a diagnosis of both anaplasmosis and LD only; 4 reported a diagnosis of just ehrlichiosis and LD only; 0 (0.00%) reported a diagnosis of just anaplasmosis and ehrlichiosis; and 2 (1.35%) reported a diagnosis of all three TBDs (Table 2). Since respondents could report more than one TBE (mean [standard deviation]: 1.68 [1.00] TBEs per respondent), the relative frequencies of TBEs by diagnostic category differs slightly from the relative frequencies of individuals. Note that since approximately two-thirds of respondents did not report a TBD diagnosis, the relative distribution of respondents and their TBEs by TBD diagnosis are easier to appreciate if undiagnosed respondents are excluded from tabulation (Table 2, Figure 1).

### 3.2. Distribution of TBEs by County-Level Endpoints

Summary counts and percentages of TBEs from each diagnostic category are provided for each county-level endpoint (Table 3A,B). County-level endpoints refer to the dichotomous “presence” or “absence” of canine TBDs, CDC LD cases, or tick presence. The diagnostic categories consist of every possible cross-tabulation of self-reported diagnoses of the three TBDs of interest (LD, anaplasmosis, and/or ehrlichiosis) as provided by survey respondents.

### 3.3. Counties with Canine TBDs

Of the 234 TBEs reported in counties with CAPC data, 178 (76.07%) occurred in counties with at least one canine anaplasmosis case, 218 (93.16%) in counties with at least one canine ehrlichiosis case, and 199 (85.04%) in counties with at least one canine LD case, and 225 (96.15%) occurred in a county with any canine TBD case (Table 3A).

Of the 157 TBEs reported by respondents with no self-reported TBD diagnosis, 120 (76.43%) occurred in counties with at least one canine anaplasmosis case, 143 (91.08%) occurred in counties with at least one canine ehrlichiosis case, 129 (82.17%) occurred in counties with at least one canine LD case, and 149 (94.90%) occurred in a county with any canine TBD case (Table 3A).

A total of 12 (92.31%) out of the 13 TBEs reported by respondents with a co-infection occurred in counties with at least one canine anaplasmosis or at least one canine ehrlichiosis case; 11 (84.62%) occurred in counties with at least one canine LD case (Table 3A).

Of the 67 TBEs reported by respondents who also reported a diagnosis of LD, 52 (77.61%) were reported in a county with canine anaplasmosis, 66 (98.51%) in a county with canine ehrlichiosis, and 64 (95.52%) in a county with canine LD (Table 3A). Due to the relatively large number of respondents who reported a TBE and an LD diagnosis (*n* = 67), we conducted a simple logistic regression to notionally quantify and compare its associations with canine TBDs. In this model, LD diagnosis was the dichotomous outcome (dependent) variable, and presence of canine LD, presence of canine anaplasmosis, and presence of canine ehrlichiosis as dichotomous predictor (independent) variables. TBEs were: significantly more likely to be reported by individuals with an LD diagnosis in counties with canine LD (OR: 6.03, *p* = 0.026); noticeably less likely (i.e., approaching significance) in counties with canine anaplasmosis (OR: 0.50, *p* = 0.095); more likely in counties with canine ehrlichiosis, albeit not significantly (OR: 2.11, *p* = 0.560).

### 3.4. Counties with CDC LD Cases

Of the 249 reported TBEs, 226 (90.76%) occurred in counties with at least one CDC-reported LD case. Of the TBEs reported by respondents with no self-reported diagnosis of LD, anaplasmosis, or ehrlichiosis, 152 (91.02%) occurred in counties with at least one CDC-Reported LD case. Of the 14 TBEs reported by respondents with a co-infection, 12 (85.71%) occurred in counties with at least one CDC-Reported LD case (Table 3B).

### 3.5. Counties with I. scapularis or I. pacificus

Of the 249 reported TBEs, 198 (79.52%) occurred in counties with either *I. scapularis* or *I. pacificus* presence, as reported by the CDC. Of the TBEs reported by respondents with no self-reported diagnosis of LD, anaplasmosis, or ehrlichiosis, 127 (76.05%) occurred in counties with either *I. scapularis* or *I. pacificus* presence, as reported by the CDC. Of the 14 TBEs reported by respondents with a co-infection, 10 (71.43%) occurred in counties with either *I. scapularis* or *I. pacificus* presence, as reported by the CDC (Table 3B).

## 4. Discussion

This study has drawn upon multi-modal data aggregated from survey respondents and national databases, with the key findings summarized in Table 3. Aimed at determining direction for potential means for detecting TBD risk, the focus of the analysis turned particularly on self-reported diagnoses and infections amongst three of the most common TBDs reported in the U.S.: Lyme disease, anaplasmosis, and ehrlichiosis.

### 4.1. Canine TBD Data as a Potential Proxy for TBD Risk

The analysis shows positive trends in the distribution of TBEs that merit further investigation. For example, other than “no diagnosis,” the largest diagnostic category was “diagnosed with LD” (*n* = 70); as would be expected, the vast majority of TBEs (>90%) reported by respondents with LD occurred in counties with CDC-reported LD cases. They were also common in counties with CAPC-reported canine LD cases as well as canine ehrlichiosis (both ~95%). A similar exploratory study in Texas found significant overlap between LD patient counties and canine ehrlichiosis [7]. A smaller proportion (~75%) occurred in counties with canine anaplasmosis. Interestingly, these frequencies amongst LD-positive respondents were similar to TBEs from “no diagnosis” respondents in counties with canine anaplasmosis (~72%), but not in counties with canine LD (~77%). In other words, a diagnosis of LD appears to be more common in those who reported a TBE in a county with canine LD or canine ehrlichiosis, but not canine anaplasmosis. These data indicate that self-reported TBEs may have validity as a proxy of county-level TBD risk via veterinary diagnostic data. Interestingly, LD-positive TBEs were less common in counties with tick presence (~87%) than in counties with CDC-reported LD cases or canine TBDs. However, this finding may be attributable to normal variance due to the relatively small sample size. Additionally, the CDC data on tick presence is not complete, but rather an ongoing report of ticks, when discovered.

Collectively, these results suggest that selected and widely available veterinary data on TBDs in canines may be sensitive to differences in TBD risk [1,8]. Thus, the canine data have value as proxies for standardized human-derived measures, such as CDC-reported LD cases which. Some amount of generalization is required, and the interpretation of related findings must consider relative differences. Nonetheless, this rapid notional analysis can help inform the design of future studies involving multimodal TBD data. Additionally, although the CAPC data is extensive, it accounts for less than a third of the estimated total canine case load. Accordingly, follow-up research will look to expand the veterinary database in light of the observations discussed above to provide a more detailed exploration of the validity of canine TBD as a potential proxy for use in statistical models of TBD risk.

### 4.2. Co-Infections and Self-Reported Diagnoses

Respondents reporting a TBE were more likely than those reporting no TBE recall to also report a diagnosis of LD or LD with co-infections. Approximately fifteen percent of LD patients who are left untreated, but present with an erythema migrans rash develop early Lyme neuroborreliosis [15]. Other reports suggest twenty-five percent of those infected with the Lyme bacteria will not develop the distinctive EM rash [16]. The implications of this finding align with medical literature suggesting that an EM rash is a valuable tool in making an LD diagnosis, particularly in endemic geographic areas, and that failure to recognize the rash may result in delayed diagnosis. 

In the present study, triangulation also provides evidence that human and canine geographic indicators may overlap. Co-infections were observed between LD and anaplasmosis and between LD and ehrlichiosis. Although no co-infections were found between just anaplasmosis and ehrlichiosis, all co-infections including both anaplasmosis and ehrlichiosis also included LD. Although data are limited, it is known that the prevalence of co-infections varies over time and by region. However, “because of small sample sizes and lack of systematic efforts to assess trends over the geographic range” of tick presence and contact, “the true prevalence of co-infections remains unknown” [8]. 

### 4.3. Integration of Survey and National Datasets

Research has shown that “self-reported human tick encounters are a robust surrogate for human tick-borne disease associated with *Ixodes scapularis* (blacklegged ticks, which are the most common LD vector) at the household and individual levels” [17]. Online surveys have the significant benefits of being able to target specific communities, assessing subjective characteristics, and—most importantly for this study—of capturing valuable epidemiological data that is otherwise inaccessible or deprioritized by major public health, scientific, and medical institutions, such as TBEs. The survey for this study was intentionally disseminated to sub-populations likely to have experienced TBEs, e.g., online support groups focused on TBDs. Spatial cluster analyses indicate that geographical approaches to determining tick presence and density may help demonstrate TBD prevalence.

## 5. Conclusions

The research presented here was conducted with the aim of developing an initial exploratory model capable of estimating TBD risk across levels of analysis via integration of multi-modal data that capture different aspects and dimensions of related disease factors and outcomes (e.g., cross-sectional surveys, official government data and reported cases, and zoonotic analogues). Given the complexity attending TBD risk and impact, model construction involved comparison of a range of iterations based on differences among variables and their interactions to identify high-value indicators and determine their influence on model fidelity. Related efforts also helped to distinguish potential gaps in knowledge and clarify further data needs and to suggest data-based optimizations for public health monitoring and research on TBDs. By establishing the presence and distribution of county-level endpoints, this study has laid the groundwork for more advanced statistical analyses and can help inform more in-depth and thorough investigations of the magnitude and characterization of TBD risk and effects. Along with tapping additional information contained in the raw county-level data, further information will be integrated and analyzed to provide valuable contextual insights and to address gaps in the database. For example, other tick species (e.g., the Lone Star Tick) can act as vectors for TBDs and are extant in different geographical locations; inclusion of such entomological data would expand the “coverage” of the study. Additionally, including counties without proper contemporaneous data could lead to outliers that disproportionately alter the results and related interpretations. Analysis of historical data—on CDC-reported LD and canine case load, as well as on tick presence and demographics, combined with older TBE information gleaned from surveys and other sources—could help elucidate trends that may not be detectable over shorter periods of time. Lastly, through the process of collecting and analyzing data across multiple sources and reports, as well as feedback from TBD sufferers, numerous opportunities can be provided to improve survey efforts and streamline data collection and applications.

## 6. Limitations

The convivence sampling sets the sample apart from the general population, and likely would be predisposed toward underestimation of TBEs, since persons who experienced a TBE with no subsequent TBD may be less likely to frequent such communities and thus less aware of and able to respond to the survey. Additionally, self-reports by voluntary respondents may be susceptible to response and cognitive biases as well as simple recollection errors. Validation challenges occur due to the inability to independently verify the survey data without direct respondent contact or transmission of sensitive personal health information or other identifiable data. Despite the survey limitations and possible sampling bias, one might say that “this is a feature not a bug”. Said another way, in order to improve the integration of “bottom-up” patient-driven and “top-down” clinical-driven data on TBDs, data collection limitations must be understood. Although data verification is not possible, respondent circumstances (i.e., burdened by TBDs) makes it difficult to imagine intentionally obfuscation or falsification of information.

Other limitations are primarily statistical. For example, tests of equality of distribution (e.g., Chi-square) were not conducted due to confounding factors (e.g., respondent-level covariates including demographics, severity of symptoms, and method of diagnosis) and the exploratory nature of this initial research. Additionally, although the time period for the canine serological data (2020) was not perfectly matched with the collection of the CDC-reported LD and survey TBE data (2000–2021), the analysis and models assumed that canine TBD test data were constant throughout 2000–2021.

## Figures and Tables

**Table 1:** Characteristics of Survey Respondents.

Age	Reported Tick Bite Encounter	Did Not Report Tick Bite Encounter	All Respondents
9–20	3	9	12
21–30	6	6	12
31–45	56	18	74
46–64	80	20	100
Older than 65	36	4	40
Age not reported	1	0	1
Total	182	57	239
Co-Infections	Recall Tick Bite	Do Not Recall Tick Bite	All Respondents
Total number of respondents who reported Lyme disease only	20	6	26
Total number of respondents who reported Lyme disease plus another infection	37	23	60
Total number of respondents who reported no Lyme disease or other infection noted	81	18	99
Total number of respondents who reported no Lyme disease but reported another infection	44	10	54
Total number of respondents	182	57	239

**Table 2:** Respondents who reported a tick bite encounter (TBE), and the number of TBEs across all respondents, by tick-borne disease (TBD) diagnostic category. Results are presented both with and without respondents who reported “no diagnosis” to better distinguish the relative distributions between different TBD diagnoses.

Diagnostic Category	Number of Respondents (% of Total)		Number of TBEs (% of Total)	
No Diagnosis/“maybe, not sure”	97 (65.54%)	-	167 (67.07%)	-
LD Only	34 (22.97%)	34 (66.67%)	56 (22.49%)	56 (68.29%)
Ehrlichiosis Only	6 (4.05%)	6 (11.76%)	10 (4.02%)	10 (12.20%)
Anaplasmosis Only	2 (1.35%)	2 (3.92%)	2 (0.80%)	2 (2.44%)
Anaplasmosis and Lyme Disease Only	3 (2.03%)	3 (5.88%)	7 (2.81%)	7 (8.54%)
Ehrlichiosis and Lyme Disease Only	4 (2.70%)	4 (7.84%)	5 (2.01%)	5 (6.10%)
Anaplasmosis and ehrlichiosis Only	0 (0.00%)	0 (0.00%)	0 (0.00%)	0 (0.00%)
Anaplasmosis, ehrlichiosis, and Lyme Disease	2 (1.35%)	2 (3.92%)	2 (0.80%)	2 (2.44%)
Total	148 (100.00%)	51 (100.00%)	249 (100.00%)	82 (100%)

**Table 3:** (**A**) TBEs (*n* = 234) in Counties with and without at least one case of canine anaplasmosis, ehrlichiosis, Lyme Disease, or any canine TBD. (**B**) TBEs (*n* = 249) in Counties with and without at least one CDC-reported Lyme Disease case, or CDC-reported presence of *I. scapularis* or *I. pacificus*.

(A)				
Self-Reported Diagnosis Category (Total Number of TBEs in Diagnostic Category)—Canine Data	Counties with 1 + Canine Anaplasmosis Cases	Counties with 1 + Canine Ehrlichiosis Cases	Counties with 1 + Canine Lyme Disease Cases	Counties with 1 + Canine TBD Cases
No Diagnoses * (*n* = 157)	120 (76.43%)	143 (91.08%)	129 (82.17%)	149 (94.9%)
Anaplasmosis (*n* = 10)	10 (100%)	10 (100%)	9 (90%)	10 (100%)
Anaplasmosis Only * (*n* = 2)	2 (100%)	2 (100%)	2 (100%)	2 (100%)
Ehrlichiosis (*n* = 15)	10 (66.67%)	13 (86.67%)	9 (60%)	14 (93.33%)
Ehrlichiosis Only * (*n* = 8)	4 (50%)	7 (87.5%)	4 (50%)	8 (100%)
Lyme Disease (*n* = 67)	52 (77.61%)	66 (98.51%)	64 (95.52%)	66 (98.51%)
Lyme Disease Only * (*n* = 54)	40 (74.07%)	54 (100%)	53 (98.15%)	54 (100%)
Anaplasmosis and Ehrlichiosis (*n* = 2)	2 (100%)	2 (100%)	1 (50%)	2 (100%)
Anaplasmosis and Ehrlichiosis Only *^,^** (*n* = 0)	No respondents reported being diagnosed with only anaplasmosis and ehrlichiosis			
Anaplasmosis and Lyme Disease (*n* = 8)	8 (100%)	8 (100%)	7 (87.5%)	8 (100%)
Anaplasmosis and Lyme Disease Only *^,^** (*n* = 6)	6 (100%)	6 (100%)	6 (100%)	6 (100%)
Ehrlichiosis and Lyme Disease (*n* = 7)	6 (85.71%)	6 (85.71%)	5 (71.43%)	6 (85.71%)
Ehrlichiosis and Lyme Disease Only *^,^** (*n* = 5)	4 (80%)	4 (80%)	4 (80%)	4 (80%)
Anaplasmosis, Ehrlichiosis, and Lyme Disease *^,^** (*n* = 2)	2 (100%)	2 (100%)	1 (50%)	2 (100%)
Total of Mutually Exclusive Coinfection Diagnostic Categories *	12 out of 13 (92.31%)	12 out of 13 (92.31%)	11 out of 13 (84.62%)	12 out of 13 (92.31%)
Total of Mutually Exclusive Diagnostic Categories **	178 out of 234 (76.07%)	218 out of 234 (93.16%)	199 out of 234 (85.04%)	225 out of 234 (96.15%)
**(B)**				
**Self-Reported Diagnosis Category (Total Number of TBEs in Diagnostic Category)—CDC Data**	**Counties with 1 + CDC-Reported Lyme Disease Cases**		**Counties with either *I. scapularis* or *I. pacificus***	
No Diagnoses * (*n* = 167)	152 (91.02%)		127 (76.05%)	
Anaplasmosis (*n* = 11)	9 (81.82%)		8 (72.73%)	
Anaplasmosis Only * (*n* = 2)	2 (100%)		2 (100%)	
Ehrlichiosis (*n* = 17)	12 (70.59%)		13 (76.47%)	
Ehrlichiosis Only * (*n* = 10)	5 (50%)		8 (80%)	
Lyme Disease (*n* = 70)	67 (95.71%)		61 (87.14%)	
Lyme Disease Only * (*n* = 56)	55 (98.21%)		51 (91.07%)	
Anaplasmosis and Ehrlichiosis (*n* = 2)	2 (100%)		1 (50%)	
Anaplasmosis and Ehrlichiosis Only *^,^** (*n* = 0)	No respondents reported being diagnosed with only anaplasmosis and ehrlichiosis			
Anaplasmosis and Lyme Disease (*n* = 9)	7 (77.78%)		6 (66.67%)	
Anaplasmosis and Lyme Disease Only *^,^** (*n* = 7)	5 (71.43%)		5 (71.43%)	
Ehrlichiosis and Lyme Disease (*n* = 7)	7 (100%)		5 (71.43%)	
Ehrlichiosis and Lyme Disease Only *^,^** (*n* = 5)	5 (100%)		4 (80%)	
Anaplasmosis, Ehrlichiosis, and Lyme Disease *^,^** (*n* = 2)	2 (100%)		1 (50%)	
Total of Mutually Exclusive Coinfection Diagnostic Categories *	12 out of 14 (85.71%)		10 out of 14 (71.43%)	
Total of Mutually Exclusive Diagnostic Categories **	226 out of 249 (90.76%)		198 out of 249 (79.52%)	

* Diagnostic Category is “mutually exclusive”; unmarked categories include any TBE with that category’s diagnosis (including co-infections), where-as each TBE belongs to only one of the “mutually exclusive” categories. Therefore, the sum of all TBEs across all mutually exclusive categories is equal to the total number of TBEs in the dataset: 249. ** Diagnostic category is a “co-infection”, one of four unique combinations of two or more TBDs: anaplasmosis, ehrlichiosis, and Lyme Disease.

## Data Availability

Data are available upon request.
